# Detection and Classification of Multi-Type Cells by Using Confocal Raman Spectroscopy

**DOI:** 10.3389/fchem.2021.641670

**Published:** 2021-04-12

**Authors:** Jing Wen, Tianchen Tang, Saima Kanwal, Yongzheng Lu, Chunxian Tao, Lulu Zheng, Dawei Zhang, Zhengqin Gu

**Affiliations:** ^1^ Engineering Research Center of Optical Instrument and Systems, Ministry of Education and Shanghai Key Lab of Modern Optical System, University of Shanghai for Science and Technology, Shanghai, China; ^2^ Department of Urology, Xinhua Hospital, School of Medicine, Shanghai Jiao Tong University, Shanghai, China

**Keywords:** Raman spectroscopy, cancer cells, SVM, LDA, QDA

## Abstract

Tumor cells circulating in the peripheral blood are the prime cause of cancer metastasis and death, thus the identification and discrimination of these rare cells are crucial in the diagnostic of cancer. As a label-free detection method without invasion, Raman spectroscopy has already been indicated as a promising method for cell identification. This study uses a confocal Raman spectrometer with 532 nm laser excitation to obtain the Raman spectrum of living cells from the kidney, liver, lung, skin, and breast. Multivariate statistical methods are applied to classify the Raman spectra of these cells. The results validate that these cells can be distinguished from each other. Among the models built to predict unknown cell types, the quadratic discriminant analysis model had the highest accuracy. The demonstrated analysis model, based on the Raman spectrum of cells, is propitious and has great potential in the field of biomedical for classifying circulating tumor cells in the future.

## Introduction

Over the past few years, there has been a gradual increase in the number of cancer deaths (Jemal et al., 2010; Ferlay et al., 2015). It be known that human organs can produce cancer cells at any stage. In fewer cases, cancer cells may develop into tumors when they accumulate to a certain extent. Circulating tumor cells (CTCs) shed from the primary tumor and spread to the peripheral blood or lymph. These CTCs are the major cause of cancer metastases and death (Fidler, 1995; Mocellin et al., 2006). Thus, it is essential to distinguish these tumor cells. Nowadays, immunocytological is still the golden standard and specific biomarkers remain the main screening method for tumor cell examination (Oosterwijk-Wakka et al., 2013; Alix-Panabières and Pantel, 2014; Bhana et al., 2015). However, some cancer cells may exhibit inadequate or vague expression of bio-markers probably by completing the epithelial-mesenchymal transition (Choueiri et al., 2013; Zerati et al., 2013; Alix-Panabières and Pantel, 2014). Besides, the use of fluorescent probes often has the disadvantage of spectral overlap and the binding of fluorescent molecules is based on the specific binding of antigen and antibody, which may change the structure of the cell molecules. It is not conducive to the follow-up of other detection experiments (Chan et al., 2008; Jones et al., 2015). Therefore, a highly sensitive, label-free, cost-efficient CTCs detection method that can accurately identify cancer cells is a pressing need.

Raman spectroscopy is a rapid and non-destructive technique for studying a biological system based on detecting molecular vibration and rotation (Krafft, 2012; Krafft et al., 2016; Popp et al., 2016; Wang et al., 2020). Numerous biochemical components can be identified by Raman spectra, for instance, genetic material, protein, and lipid, all have their unique peaks in the Raman spectrum (Stone et al., 2004; Brauchle et al., 2014; Fang et al., 2019). As a well-known fact, mutations in cancer cells are always accompanied by wireless proliferation, which leads to a huge increase of genes in the nucleus. Some cancer cells also suffer protein changes on the cell membrane or accumulate large amounts of lipids in the cytoplasm (Abramczyk et al., 2009; Yue et al., 2014; Surmacki et al., 2015). At present, there have been many studies reporting the use of Raman microspectroscopy combined with multivariate statistical analysis methods to distinguish and classify cell types (Kong et al., 2015). Machine learning-based analysis methods such as support vector machine (SVM), principal component analysis (PCA), linear discriminant analysis (LDA), and quadratic discriminant analysis (QDA) were introduced in combination with Raman spectroscopy which has successfully attained the identification of many cells and tissues in the declining years (Dixon and Brereton, 2009; Neugebauer et al., 2010; Pudlas et al., 2011; Zhang et al., 2016; Siqueira et al., 2017). However, these research only compare tumor cells with normal cells produced by the same organ, the differences between tumor cells from different organs are not clear (Crow et al., 2005; Krishna et al., 2005; Chan et al., 2008; Pijanka et al., 2013). We expect to get a broader spectrum of cancer cells in order to recognize CTCs at an early stage, which will help to identify the diseased organs in advance, allowing cancer patients to avoid mortality outcomes. The accuracy and classification characteristics using the statistical analysis methods for cell samples also need to be verified by comparison.

In this article, we chose the cell samples from kidney, lung, liver, breast, and skin as the research object, and explore the process of living cell detection by confocal Raman spectroscopy. With the combination of multivariate statistical methods, Raman spectra of various types of cells are classified based on three models i.e. SVM, LDA, and QDA, the accuracy of classifying the accurate cell types is above 95%. In order to verify the feasibility of the model, the results validate that high sensitivity is realized to predict the unknown cell types. Furthermore, the comparison of the pros and cons of the three models is also discussed. The flexible combination of Raman spectroscopy and various modeling methods can immediately identify a variety of cells at a high accuracy which will provide potential applications in CTCs detection.

## Methods and Experiments

### Cell Culture and Sample Preparation

Cells in this study are obtained from the American Type Culture Collection (ATCC), which is listed in Table 1. All the cells were grown in Dulbecco's Modified Eagle’s Medium (DMEM) with 10% fetal bovine serum (FBS) and antibiotics (penicillin and streptomycin) at 37°C in a humidified atmosphere of 5% CO_2_. Cells in the logarithmic phase were taken to harvest with Trypsin-EDTA and suspended in phosphate-buffered saline (PBS 1×) before Raman measurement. All cells were collected under the same condition. About 2 ml of the cell suspension was added into a culture dish with a quartz glass bottom. Approximately three-quarters of the cells were used as training and validation, while another quarter was for the prediction set.

**TABLE 1:** Names of cell lines and cell numbers.

Cell lines	Cell names	Total number	Calibration	Prediction
786-O	Human clear cell renal cancer cells	52	40	12
HKC	Human kidney tubular epithelial cells	52	40	12
HepG-2	Human hepatoblastoma cells	38	29	9
A549	Human non-small cell lung cancer cells	57	45	12
A375	Human malignant melanoma cells	56	44	12
4T1	Mouse breast cancer cells	50	38	12

### Raman Spectroscopy Measurement

Raman spectra of living cells were obtained from a laser confocal Raman spectrometer (RAMANtouch, Japan) with 532 nm laser excitation. Before the Raman test, a CCD detector must be cooled to −70°C, and the system was calibrated by using silicon with its peak at 520.5 cm^−1^. The laser was focused by a ×60 water immersion objective lens (NA = 1.0, Nikon, Japan) onto the sample. To ensure an appropriate signal-to-noise ratio without damaging the samples, the laser power was controlled at about 10 mW. Single point measurement mode was used and the laser spot size on the sample was about 0.65 µm. The exposure time for each cell was kept at 8 s for each time and tested three times. Subsequently, the spectrometer automatically took the averaged spectra. The test range was in the Raman low wavenumber region and a minimum of 35 spectra were obtained from each kind of cell. Regions of each cell’s nucleus were preferentially sampled. The measurements of all cells were worked out under the same conditions.

### Data Processing and Analysis

Raman spectral data were pre-processed using WiRE 4.2 (Renishaw, United Kingdom), with baseline corrected and smoothed, and cosmic rays were removed if existing. Before analysis, the Raman shift in each spectrum was cut into the ‘fingerprint’ region from 600 to 1800 cm^−1^, which removed the Raman peak of the quartz glass substrate. The Raman intensity was normalized and unified as the relative intensity of arbitrary unit (a.u.) using OriginPro 9.1. (OriginLab Corp., Northampton, MA, United States) (Zhao et al., 2007; Zhang et al., 2010a; Zhang et al., 2010b). All spectral data were corrected for baseline translation and shift phenomena using the EMSC (extended multiplicative signal correction) algorithm, assuming the average of all spectral data as the ideal spectrum(Popp et al., 2018). Multivariate statistical analysis methods i.e. SVM, PCA, LDA, QDA were carried out with MATLAB R2016b (MathWorks, Inc., United States) and The Unscramble@10.4 (CAMO, Oslo, Norway).

## Results and Discussion

**FIGURE 1:**
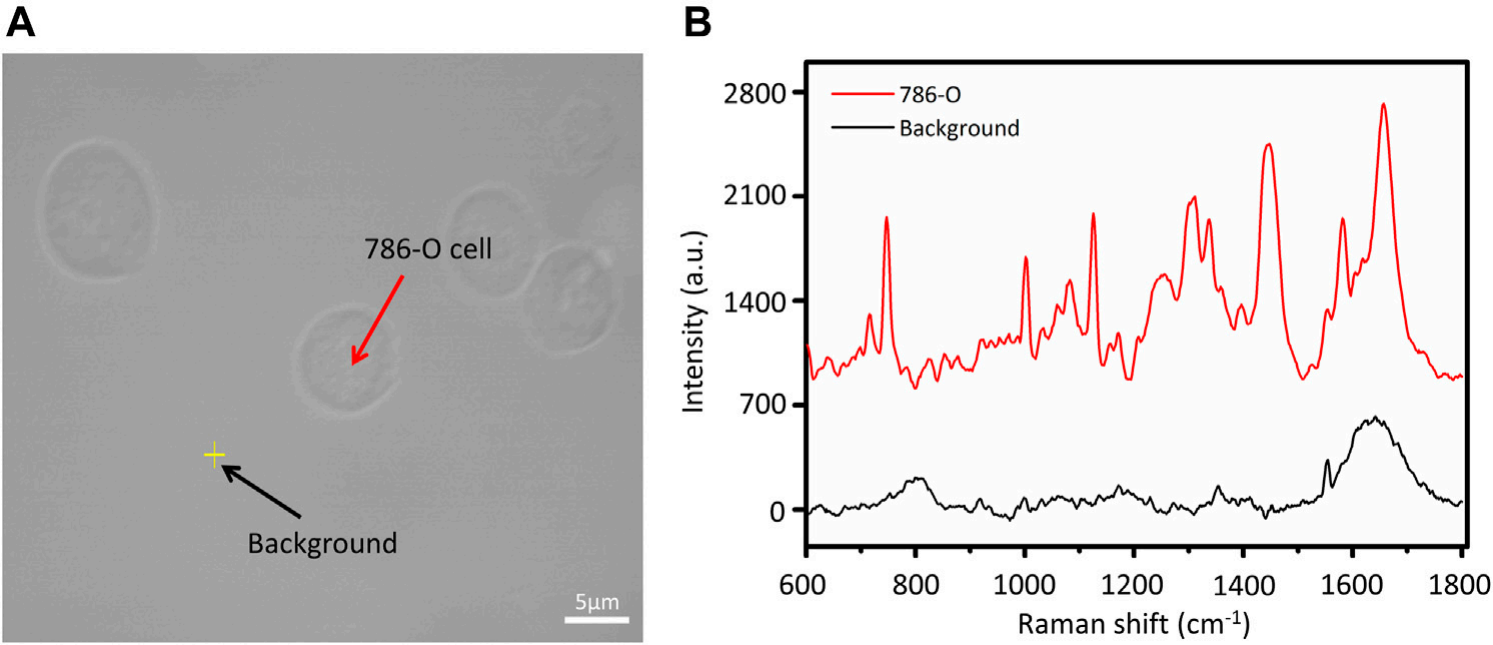
**(A)** Bright field image of 786‐O cells and **(B)** Raman spectrum of background and cell.

To determine whether the spectral data obtained from the measurement were Raman signals of cells, we checked the signals around the cells, the laser point was slightly above the bottom to focus on the cells. Figure 1A shows the bright field image of a typical 786-O cell and the blank background near the cells, while Figure 1B shows the Raman spectra of the two corresponding points. Unlike the cell curve, deprived of a large number of sharp Raman peaks, the background curve is relatively smoother. The only background peak displayed in the spectrum was at about 1554 cm^−1^. It demonstrates that the Raman signal in quartz glass background exhibited extremely low interference which is consistent with the description in the previous report (Palonpon et al., 2013).

**FIGURE 2:**
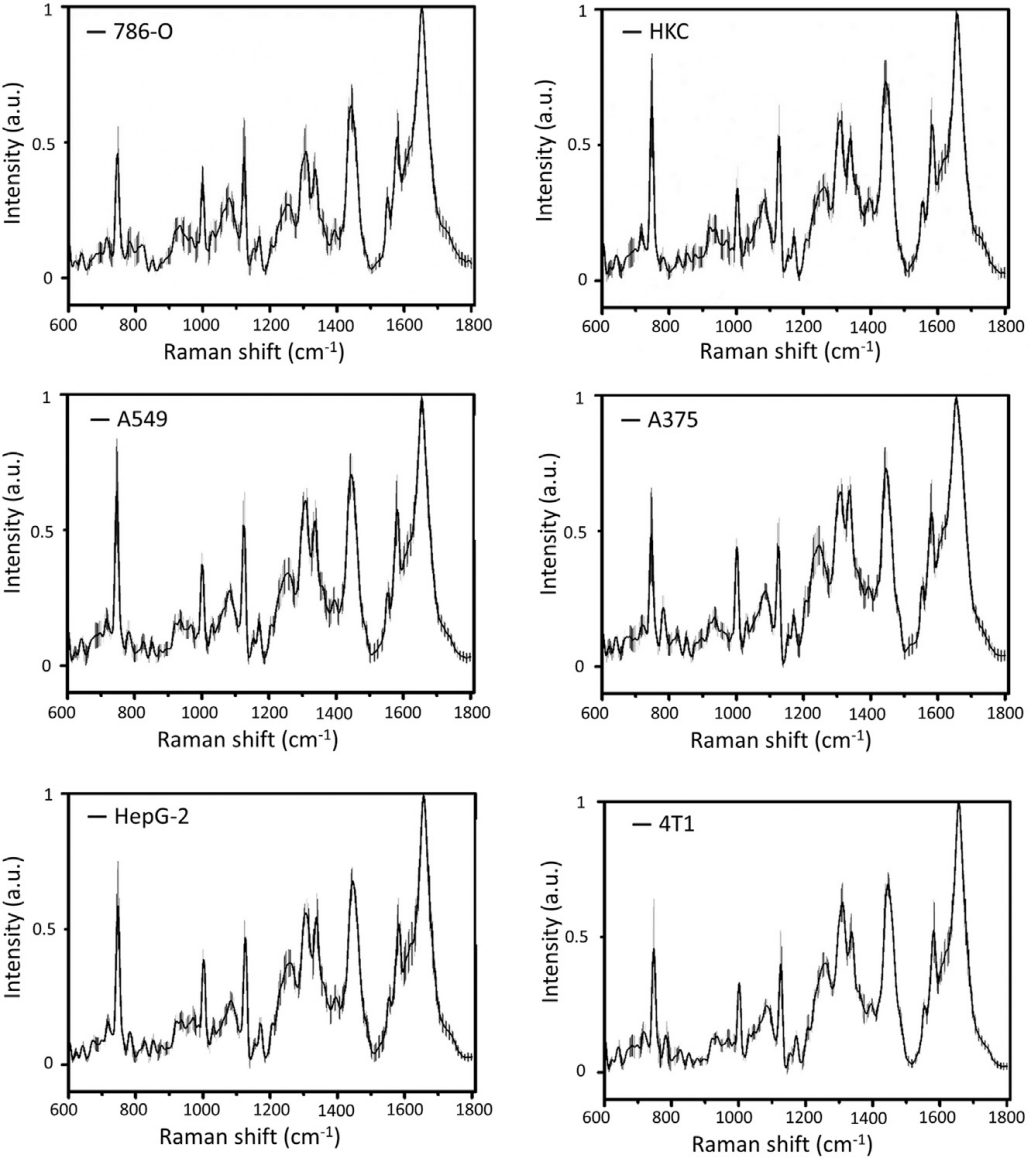
The average Raman spectra of 6 kinds of cells with error bars.

Figure 2 manifests the average Raman spectrum curves of six types of cells. The shadow area is the error bars which represent the standard deviation of the mean value. The main common Raman peaks at 642, 831, 851, 1171, 1208, 1604, 1620 cm^−1^ (tyrosine), 1003 cm^−1^ (phenylalanine), 1246 cm^−1^ (Amide III), 1337 cm^−1^ (tryptophan), 1655 cm^−1^ (Amide I) are assigned to proteins. Strong and wide peaks are corresponding with lipid at 1124 cm^−1^ (C–C stretching mode), 1252 cm^−1^ (=CH in-plane bending), and 1445 cm^−1^ (CH2 deformation). Other bands at around 747 cm^−1^ (thymine), 1176 cm^−1^ (cytosine, guanine), 1311 cm^−1^ (adenine), 1581 cm^−1^ (pyrimidine ring of nucleic acids) are assigned to DNA and RNA. Details of Raman peaks assignment to cell spectrum are presented in Table 2 (Stone et al., 2004; Notingher and Hench, 2006; Wood et al., 2007; Surmacki et al., 2015). Various cells have quite comparable Raman spectra due to the similar biochemical components (as shown in Figure 2). But there are also a few Raman peaks that are different, such as distinct peaks at 831 and 850 cm^−1^, which represent tyrosine residue conformations (Zhuang et al., 2013). These spectral peaks distinguished by the human eye may not be sufficient to accurately identify different cells. Herein, we used a multivariate statistical analysis algorithm (SVM, PCA, LDA) to process the data set and analyze the subtle differences of the Raman spectrum among different cells.

**TABLE 2:** The representative peak assignments in cell Raman spectra (Stone et al., 2004; Notingher and Hench, 2006; Wood et al., 2007; Surmacki et al., 2015).

Raman shift (cm^−1^)	DNA	Protein	Lipid
642		Tyrosine (C–C)	
747	Thymine		
831/851		Tyrosine	
1003		Phenylalanine	
1124			C–C stretching mode
1176	Cytosine, guanine	Tyrosine	
1208		Tyrosine	
1252		Amide III	=CH in-plane bending
1311	Adenine		
1337	Adenine, guanine	Tryptophan	
1445			CH2 deformation
1581	Pyrimidine ring		
1604		Tyrosine	
1620		Tyrosine	
1655		Amide I	

**FIGURE 3:**
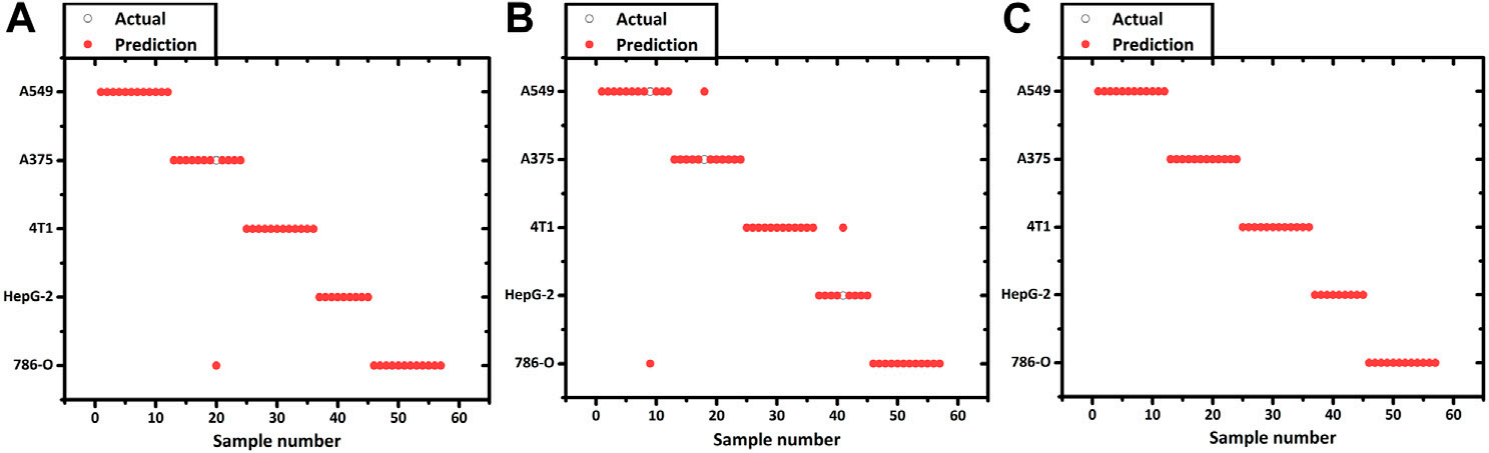
Prediction result of 5 kinds of cancer cell lines using the **(A)** SVM, **(B)** LDA and **(C)** QDA model.

SVM is a supervised learning method for the binary classification of data. It finds the maximum geometric margin hyperplane between two kinds of learning data sets and using the optimal hyperplane to distinct the two data sets into two sides to complete the classification (Dixon and Brereton, 2009; Zhang et al., 2016). In this paper, we use an SVM with a linear kernel function to realize the discriminative classification of prediction samples. To prevent overfitting, while ensuring the classification accuracy, the penalty factor *C* selection was set to 1. Each spectrum was cross-validated with 10 segments. Initially, we performed a feasibility validation using cancer and normal cells derived from the same organ which had an apparent difference of biochemical components. 40 cancer cells (786-O) and 40 normal cells (HKC) were used to construct the SVM model. Meanwhile, to verify the accuracy of the SVM model for predicting unknown cells, a set containing 24 new cells (12 786-O, 12 HKC) was used. The prediction accuracy was 100% so that the cancer cells and normal cells could be distinguished from each other. The details of predicting results are shown in Table 3. Using the same SVM method could additionally construct a classification model among five different cancer cells. 196 cancer cells are used to form a training set (including 40 786-O, 29 HepG-2, 45 A549, 44 A375, 38 4T1) to construct the model. Table 4 shows the details in a confusion matrix of the SVM classification model and the validation accuracy in the training set was 100%. However, the predictive performance needs to be tested. The prediction rate of these SVM models is verified by a prediction set of 57 new ‘unknown’ cells (actually already known but not included in the training set, including 12 786-O, 9 HepG-2, 12 A549, 12 A375, 12 4T1). The result is shown in Figure 3A and the prediction of accuracy is 98.25%.

**TABLE 3:** Prediction result of cancer cells/normal cells with SVM classification.

	Actual set	
Prediction set	786-O	HKC
786-O	12	0
HKC	0	12

**TABLE 4:** Confusion matrix of five cancer cell lines using the SVM classification model.

		Actual sets				
		A549	A375	HepG-2	4T1	786-O
Prediction set	A549	45	0	0	0	0
	A375	0	44	0	0	0
	HepG-2	0	0	29	0	0
	4T1	0	0	0	38	0
	786-O	0	0	0	0	40

Although SVM had successfully established the classification among different cancer cells, it has the drawbacks of a complex and time-consuming model. SVM is a binary classifier, seeking the optimal hyperplane between the two data sets. While dealing with the problem of multi-sample classification, SVM models should be constructed between every two samples. As for *N* different types of cells, at least *N* × (*N*-1)/2 decision values should be considered. While dealing with two distinct cell types (cancer cell 786-O and normal cells HKC, the *N* is not too large), the training speed is relatively swift. However, to deal with the classification of multiple cancer cells, the number of binary classifiers increases as a quadratic function concerning *N*, which significantly increases the amount of training operation and reduces the training speed (Dixon and Brereton, 2009). Therefore, we employ an LDA method to predict and classify various cancer cells. LDA is a classical linear supervised learning method to reduce the dimension and classify, which has been reported in classification of cancer Raman spectra(Dochow et al., 2011; Pijanka et al., 2013). Given a labeled set of training samples, LDA tries to project the samples into low-dimensional space, so that the projection points of the same samples are as close as possible and the projection points of the heterogeneous samples are as far as possible. After projection, the different types of the sample will be distributed in different regions of the lower dimensional space, and the prediction sets will also be projected in the space according to the previously calculated dimensionality reduction rules. Afterward, the category of the new sample is determined based on the location of the projection point (Dixon and Brereton, 2009; Siqueira et al., 2017).

**FIGURE 4:**
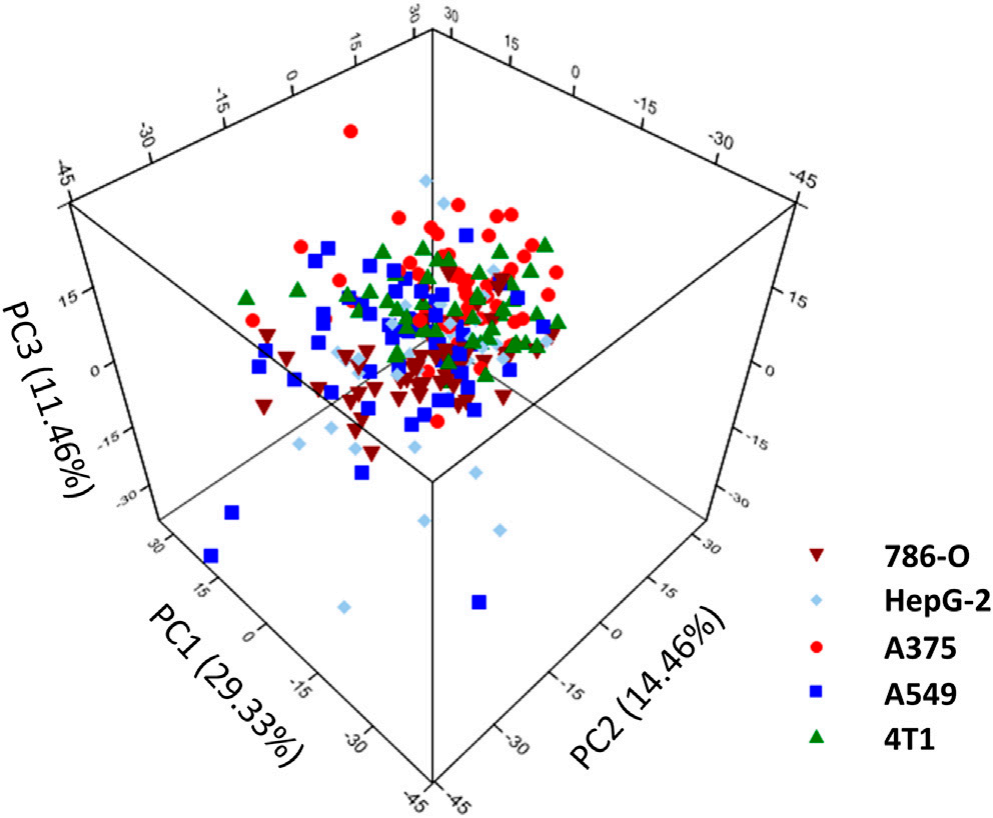
The top three PCs’ score plot of different cancer cells.

**FIGURE 5:**
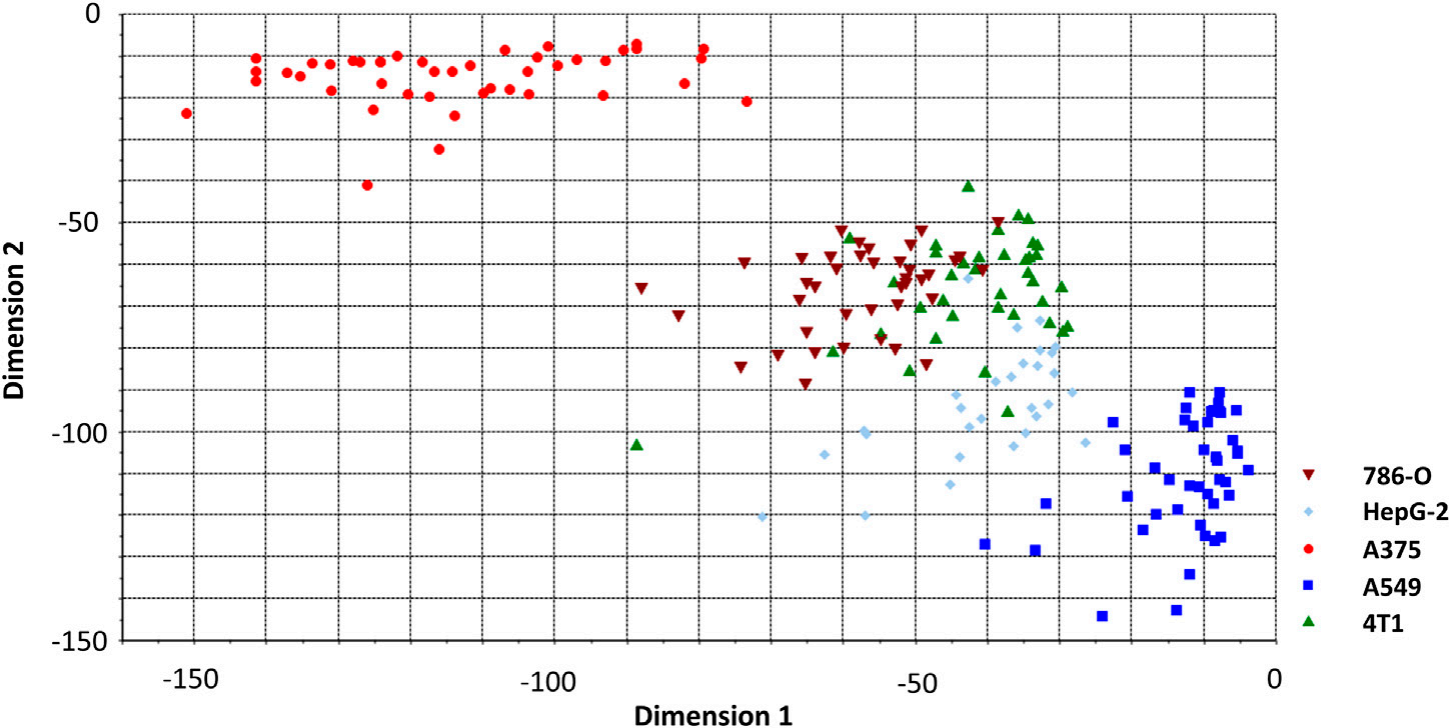
LDA classification model classifying spectrum of cancer cell lines. Two of five dimensions are plotted.

Before constructing the LDA and QDA classification model the Raman spectral data needs further process due to a large number of variables arrays. PCA is introduced to eliminate any overlapped information in the spectrum through a multivariate linear transformation which extracts the eigenvalues of the data matrix and then reconstructs a basic eigenvector to form a new data set (Dixon and Brereton, 2009). Through the transformation, PCA could also classify some simple data sets. However, in this study, PCA is not implemented with the SVM model. Various studies have directly used the SVM method to analyze the Raman spectrum. We suppose that this is because the SVM method can better solve the problem of classification of high-dimensional data and there is no need of reducing the dimension of the data in advance. According to the validation on our spectrum dataset, the accuracy of PCA + SVM trained and predicted is indeed lower than the method that uses SVM directly. In this contribution, Raman shift in the spectrum was including 683 variables evenly distributed over the region of 600–1800 cm^−1^. The 197 cancer cell spectrum, previously used to build SVM models, is still used as the verification set here. Figure 4 shows the result of three dimensions of the first principal component (PC1, PC2, and PC3). The five groups of cells were spatially clustered but could not be well separated. This shows that the classification effect of PCA is not ideal when dealing with high-dimensional data with complex and fuzzy noise distribution. The comprehensive contribution rate of three PCs was 55.25%, which represented the main variances. Due to the more PC numbers retains more original Raman spectrum information (Tang et al., 2017), to improve the accuracy of subsequent predictions, we increased the number to the first 20 PCs, which described 85% of variables. Subsequently, the LDA model was constructed with these PCs. The prior probabilities were calculated from the training set. Cell spectral data was classified in fivefold dimensional space. Figure 5 selects the projection of two dimensions to plot, which represents the division of A375 (red) and A549 (blue). Under the combination of any two different dimensions, the discrimination of the two types of cells can be found. The prediction result is indicated in Figure 3B with an accuracy of 94.73%, which is a little lower than the SVM model.

**FIGURE 6:**
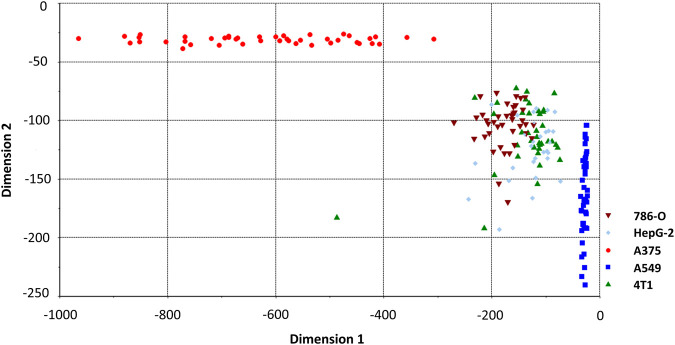
QDA classification model classifying spectrum of cancer cell lines. Two of five dimensions are plotted.

We perceived that some cell spectra were misclassified into the wrong category. It is due to the limitation of LDA which is linearly partitioned, while the boundary of the cell Raman spectrum is irregular. QDA is based on LDA which uses quadratic information and far higher complexity hypersurface to enhance the accuracy of classification (Dixon and Brereton, 2009; Tang et al., 2017). Figure 6 shows a QDA analysis model using two of the five dimensions. Utilizing the verification set previously constructed, the 57 unknown cell spectrum were also used for QDA. Figure 3C presents the prediction detail. Each cell spectra had precisely predicted with a 100% prediction accuracy. Finally, we compared the efficiency of building these models. The computation time to construct the SVM model costs 0.47 s, while the LDA and QDA method was 0.014 and 0.018 s. SVM costs the longest time, which is consistent with the previous theory. Constructing the QDA model is slightly slower than LDA, but both are in the same order of magnitude. Consequently, the QDA model had the elite classification and prediction ability with relatively good efficiency.

In practical applications (such as CTC detection), besides the tumor cells, another cellular material is contained in the peripheral blood. Mainly including leukocytes, red blood cells et al. Since the supervised learning model is used, we need to know the extent possible about the cell types that may be contained in the samples. Relative to the multivariate cancer cells, these haemocytes have a greater difference in size, morphology, components, and are easier to distinguish. By the existing devices, such as magnetic bead sorting or optical tweezers, the tumor cells can be preliminarily distinguished from these common haemocytes cells (Dochow et al., 2011; Rao et al., 2018; Chang et al., 2020). To avoid haemocytes, that are not completely excluded in the actual detection affecting the discrimination accuracy, the Raman spectrum of haemocytes could also be added to the training database. Some of the previous studies, for example, Zhang et al. successfully distinguished cancer cells from leukocytes using Raman spectroscopy(Zhang et al., 2016). For clinical applications, the analytical throughput of cells is also an important indicator. The time spent for each cell is 24 s. Considering a little more consumption of time by the spectrometer to automatically adjust the laser spot to switch to the other cells, the Raman test throughput was <150 cells/h. Usually, there are several CTCs out of 10^3^–10^7^ nucleated cells in a patient's blood sample (Pachmann et al., 2005). To meet the need for clinical application in the future, we can pre-dispose of tumor cells by magnetic bead enrichment before Raman spectroscopy measurement, with the number of tumor cells enriched being much smaller than this test throughput. Therefore, it is acceptable to measurement within an hour, which holds a great prospect for rapid detection in the future.

## Conclusion

In this study, Raman spectra of six different cells were obtained from confocal Raman spectroscopy. Different cell lines had tiny different spectra. The Raman peaks of 831 and 850 cm^−1^ showed the tyrosine residue conformation, which revealed the difference of renal and breast cancer cells from the liver, lung, and skin cancer cells. By using multivariate statistical methods of SVM, LDA, and QDA, we further studied the spectral differences between various cancer cells. The identification accuracy and advantages were compared to discuss. The SVM and LDA model had identical specific accuracy of classifying and predicting cell spectrum, but LDA is more beneficial when the type and number of samples are vast. QDA is a variant of LDA and had a better sensitivity of 100% prediction accuracy in the analysis of cellular Raman spectra. In the follow-up study, we will accumulate more cell spectrum to improve the reliability of the calibration sample library and implement it to identify the circulating tumor cells in the peripheral blood of tumor patients. Raman spectroscopy is a powerful, rapid, and non-destructive means in the identification of biochemical components. It has the potential to play a substantial role in the detection of cancer metastasis in an early stage of cancer or after surgery in the future.

## Data Availability

The raw data supporting the conclusion of this article will be made available by the authors, without undue reservation.
